# Transcriptome-Wide Identification and Development of SSR Markers for Genetic Diversity Studies in Medicinal *Polygonatum* Species

**DOI:** 10.3390/ijms27062632

**Published:** 2026-03-13

**Authors:** Wenjuan Huang, Hui Wang, Majin Yang, Changhua Ye, Zhen Li, Shengfu Zhong

**Affiliations:** 1 Sichuan Academy of Agricultural Characteristic Plants, Chengdu 611730, China; hwjuan@scsaas.cn (W.H.); wanghui@scsaas.cn (H.W.); yangmj93@scsaas.cn (M.Y.); ye@scsaas.cn (C.Y.); lizhen@blooge.cn (Z.L.); 2 Industrial Crops Research Institute, Sichuan Academy of Agricultural Sciences, Chengdu 610300, China

**Keywords:** Polygonati Rhizoma, germplasm resource, simple sequence repeats, molecular marker, genetic diversity analysis

## Abstract

The genus *Polygonatum* encompasses numerous species with complex phenotypes, necessitating robust molecular markers for accurate species identification and superior germplasm screening. This study identified and developed SSR markers based on transcriptome analysis of three *Polygonatum* species to assess the genetic diversity of *Polygonatum* resources. The results showed that a total of 43,217 SSR loci were detected, and 31,703 primer pairs were successfully designed. Characterization of SSR motifs revealed mono-nucleotide repeats (SNRs) were the most frequent (59.45%). Unigenes containing SSRs were annotated across seven databases. In KEGG, 222 pathways were assigned, with genes annotated to carbohydrate metabolism being the most abundant. To validate and apply these markers, 100 primer pairs covering all eight SSR locus types were tested across 21 *Polygonatum* accessions. Of these, 49 polymorphic markers were identified, revealing high genetic diversity, with average expected heterozygosity (He) and polymorphism information content (PIC) values of 0.763 and 0.718, respectively, alongside significant population differentiation (Fst = 0.307). Cluster analysis grouped 21 accessions into three groups, which correlated with certain agronomic traits. Nine core markers were selected that effectively distinguished six species and intraspecific groups. Notably, the FB-9 marker, associated with polysaccharide biosynthesis, effectively discriminated among six *Polygonatum* species and also distinguished distinct germplasm resources within *P. kingianum* var. *grandifolium*. Overall, the transcriptome-derived SSR markers validated in this study constitute valuable resources for gene function analysis, population genetics research, and variety identification and genetic improvement of *Polygonatum*.

## 1. Introduction

Polygonati Rhizoma (PR), as a traditional Chinese medicine with dietary purposes, is derived from multiple botanical origins within the *Polygonatum* genus. These include *Polygonatum sibiricum* Hua (PS), *P. kingianum* Col et Hemsl. (PK), *P. cyrtonema* Red. (PC), *P. kingianum* var. *grandifolium* D. M. Liu & W. Z. Zeng (PKG) and *P. cirrhifolium* (Wall.) Royle (PCI) [1,2,3]. In this paper, the authors collectively refer to them as medicinal *Polygonatum* species (MPs). The genus *Polygonatum* comprises perennial herbaceous plants, that are extensively distributed throughout Asia [4,5]. These plants are frequently utilized as traditional Chinese medicinal resources and nutritional foods [6,7]. PR contains bioactive components such as flavonoids, polysaccharides, saponins, and alkaloids [8]. Traditionally, it is recognized for its functions in tonifying the middle jiao (spleen-stomach digestive center), replenishing qi (body’s fundamental vital energy), strengthening the spleen (whole digestive system, partial blood, immune, neuroendocrine, and motor systems), nourishing the lungs (respiratory system and partial digestive, circulatory and urinary systems), and tonifying the kidneys (reproductive, urinary, nervous, and skeletal systems) [6]. Contemporary pharmacological studies have demonstrated its effects in lowering blood sugar, modulating gut microbiota, enhancing immunity, combating fatigue, improving memory, and exhibiting antioxidant activity [8]. Long-term consumption is believed to promote bodily lightness, extend lifespan, and prevent hunger, thereby showing great potential in addressing both explicit hunger and hidden hunger [9,10]. Thus, it has become one of the most rapidly developing traditional Chinese herbs in recent years.

The genus *Polygonatum* exhibits significant morphological variation and admixture among cultivars [11,12,13,14,15]. This makes accurate species identification based solely on phenotypic characteristics challenging, resulting in uneven quality and potential impacts on medication safety. For instance, *P. odoratum* (Mill.) Druce (PO), a traditional Chinese herb Polygonati Odorati Rhizoma (POR) with distinct therapeutic properties, is difficult to distinguish from PC due to their similar alternate phyllotaxis and leaf morphology [11]. Similarly, PKG exhibits polyploidy and aneuploidy in natural populations. Its leaves can display alternate, whorled, or irregular arrangements across different growth stages, with variable leaf shapes, further complicating its distinction from other *Polygonatum* species [4]. Additionally, the majority of *Polygonatum* species experience senescence of their aerial parts in winter, with rhizomes entering dormancy. Identification during this phase depends exclusively on the morphological characteristics of the rhizomes [15]. However, rhizome morphology is subject to environmental influences and exhibits considerable variability. For example, distinguishing between PC and PO, or PC and PKG based solely on rhizome traits is highly challenging, labor-intensive, and occasionally unfeasible [12,13,14]. Therefore, to effectively protect MP resources, it is imperative to conduct systematic genetic diversity analysis and develop corresponding molecular markers. These efforts are essential for germplasm and variety identification [11].

Simple Sequence Repeat (SSR) markers require only small amounts of plant material and offer advantages in speed, efficiency, and precision. Consequently, they have been widely developed and applied in numerous species [16,17,18,19]. For example, Li et al. used 15 SSR markers to assess the genetic diversity of 25 *Ilex asprella* resources, classifying them into three groups [17]. Yu et al. identified six SSR loci associated with salt-tolerance genes based on transcriptome data and classified four alfalfa varieties based on different salinity tolerance [18]. Zhang et al. analyzed the genetic diversity and relationships of 44 hexaploid *Camellia oleifera* cultivars using 30 markers, determined a minimal two-marker set capable of distinguishing all tested cultivars, and constructed cultivar dosage-aware SSR (DA-SSR) fingerprints [19]. Currently, research on genetic diversity analysis and molecular marker-assisted breeding for *Polygonatum* germplasm resources is relatively limited, which restricts the development and application of SSR markers for species identification in MPs. Hence, it is imperative to develop efficient and species-specific molecular markers to facilitate germplasm characterization and ensure the precise identification of MPs.

Transcriptome sequencing serves as a primary source for SSR molecular markers. Studies indicate that expressed sequence tag-derived simple sequence repeat (EST-SSR) markers outperform inter-sample sequencerepeat (ISSR) markers in metrics such as gene diversity, polymorphic information content, heterozygosity identification, and marker specificity [20]. As EST-SSRs are derived from transcribed regions, the expressed portions of the genome, and they are likely to have greater functional relevance and may be directly associated with phenotypic traits [21,22]. Therefore, we propose that SSR markers derived from transcriptome data can serve as effective tools for elucidating the genetic diversity of MPs and addressing the difficulties in species identification arising from morphological similarities. Accordingly, the present study was designed to conduct transcriptome sequencing on three MPs (*P. kingianum*, *P. cyrtonema*, and *P. kingianum* var. *grandifolium*) with the following aims: (1) to develop and characterize polymorphic SSR markers and analyze their distribution across the different species; (2) to assess the genetic diversity and phylogenetic relationships among 21 *Polygonatum* accessions, and to determine their clustering patterns; and (3) to identify core primer sets capable of reliably and accurately discriminating among MPs and their potential adulterants. The findings provide a scientific foundation for the conservation, breeding, and quality control of MPs.

## 2. Results

### 2.1. Identification of SSRs

A total of 198,752 unigenes, with a total nucleotide length of 142,330,816 bp (Table S1), were obtained from transcriptome sequencing of rhizomes from three MP species. Subsequently, SSR motifs were identified in these unigenes, and their characteristics were compiled (Table S2). 47,287 SSR motifs were identified, distributed across 36,842 SSR-containing unigenes, which accounted for 18.54% of all unigenes. The SSR frequency was 23.79%, corresponding to the number of SSRs per unigene. Among these, 5522 unigenes (14.99% of SSR-containing unigenes) contained two or more SSR loci, and 2918 unigenes harbored two or more SSR motif types.

### 2.2. Frequency and Distribution of SSRs in Three MPs

#### 2.2.1. Composition and Characterization of SSRs Based on Motifs

**Figure 1:**
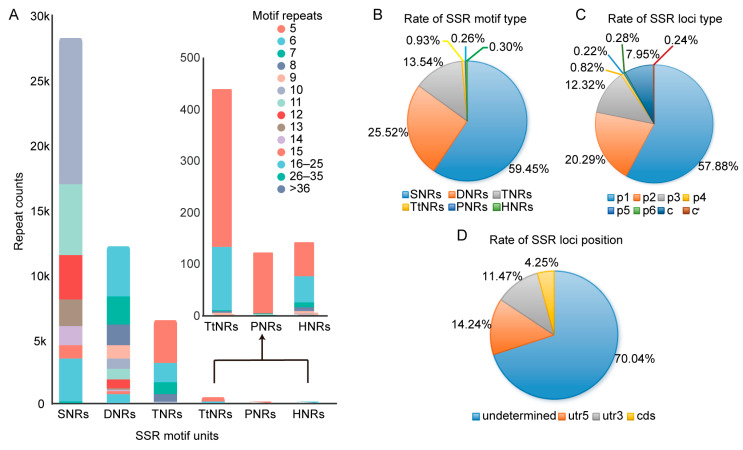
Characteristics of SSR loci and motifs identified from the transcriptomes of three MPs. (**A**) Frequency of unigenes containing different numbers of SSR loci; (**B**) Proportion distribution of different SSR motifs; SNRs, mono-nucleotide repeat types; DNRs, di-nucleotide repeat types; TNRs, tri-nucleotide repeat types; TtNRs, tetra-nucleotide repeat types; PNRs, penta-nucleotide repeat types; HNRs, hexa-nucleotide repeat types; (**C**) Types and proportions of SSR loci; p1, mono-nucleotide repeat type; p2, di-nucleotide repeat type; p3, tri-nucleotide repeat type; p4, tetra-nucleotide repeat type; p5, penta-nucleotide repeat type; p6, hexa-nucleotide repeat type; c, complex repeat type; c*, interrupted complex type; (**D**) Frequency of SSR locus positions within unigenes.

SSR motif analysis identified all repeat types, ranging from mono-nucleotide repeats (SNRs) to hexa-nucleotide repeats (HNRs), with 141 distinct repeat unit compositions (Figure 1A, Table S3). SNRs were the most abundant, comprising 59.45% (28,114) of total SSRs, followed by 12,068 di-nucleotide repeats (DNRs) (25.52%) and 6404 tri-nucleotide repeats (TNRs) (13.54%). Penta-nucleotide repeats (PNRs) (33) were the least frequent, representing only 0.26% of all SSRs (Figure 1B). Among SNRs, A/T repeats dominated (99.47%). In DNRs, AG/CT was the most common (16.73%), while CG/CG was the rarest (0.13%). For TNRs, three motifs exceeded 2% frequency: AAG/CTT, AAT/ATT, and AGG/CCT (Table S4).

#### 2.2.2. Composition and Characterization of SSR Loci

Based on SSR loci analysis, SSRs were classified into eight types. In addition to the simple repeat types p1 to p6, compound microsatellites were identified and further divided into two complex repeat types: c and c* (Figure 1C). Type c consists of two or more repeat units directly adjacent to each other, whereas type c* consists of repeats interrupted by non-repeat sequences. Among the simple repeat types, p1 was the most abundant representing 57.88% of all SSR loci, while p5 was the least, accounting for only 0.22%. 3539 SSR loci were identified as complex repeats, with type c repeats comprising the majority (97.03%) (Table S5). Position analysis revealed that 4.25% of SSR loci were located in the coding sequence (CDS) regions (Figure 1D).

#### 2.2.3. Characterization of TNRs

The detection of TNRs in expressed sequences can directly alter gene coding sequences, thereby affecting the corresponding gene expression products and potentially leading to phenotypic changes [23]. In this study, 60 TNRs (excluding AAA, TTT, CCC, and GGG) were identified in the rhizomes of *Polygonatum* plants. These TNRs in the p3 types were distributed across 5060 unigenes, harboring 5323 SSR loci. Among these, 1324 unigenes contained TNRs within CDS. The TNRs can be translated into 20 different amino acids. The five most frequent amino acids encoded were leucine, serine, isoleucine, alanine, and asparagine, with 790, 505, 384, 381, and 348 loci, respectively (Figure S1). Furthermore, 227 unigenes contained TNRs encoding two or more different amino acids (Table S2).

### 2.3. Functional Annotation of Unigenes Containing SSRs

**Figure 2:**
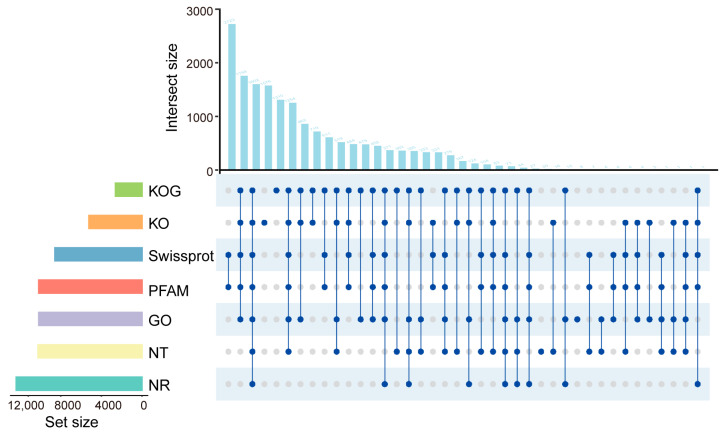
Venn diagram of 7 annotation database. The numbers on the top bar represent the results of the intersection of the databases in the matrix below corresponding to the databases with blue dots, and gray dots indicate databases without intersection. The bars on the left represent the number of unigenes fully annotated to each database.

Multiple databases (NR, KOG, GO, SwissProt, NT, Pfam and KEGG) were used to annotate the 36,842 unigenes containing SSRs (Figure 2). 17,086 unigenes (46.83%) were annotated in at least one database; 1602 unigenes (4.35%) were successfully annotated in all seven databases. Annotation against the NR database annotated 12,383 unigenes (33.61%), a notably higher proportion than any other database. In the NR annotations, the species most frequently matched was *Asparagus officinalis*, accounting for 52.75% of NR-annotated sequences, followed by *Iris pallida* (2.84%) and *Phoenix dactylifera* (1.20%) (Figure S2A). For other databases, the annotation rates for GO, NT, Pfam, and SwissProt each exceeded 20%, ranging from 23.26% to 27.78%. In contrast, the annotation rates for KEGG and KOG were lower, accounting for only 14.18% (5224 unigenes) and 7.08% (2610 unigenes), respectively (Figure 2).

GO annotation assigned 4346 distinct functional terms to all unigenes containing SSRs. These terms were grouped into three major categories: cellular component (three subcategories), molecular function (22 subcategories), and biological process (16 subcategories) (Figure S2B). Within the biological process category, ‘cellular process’ was the most abundant (61.86%), while ‘intraspecies interaction between organisms’ was the least frequent (0.08%). In KEGG, 5025 unigenes were annotated and mapped to 222 pathways, which were classified into seven major categories (Figure S2C). Among these categories, Brite Hierarchies (containing 3801 unigenes in 46 pathways) and Metabolism (containing 1219 unigenes in 112 pathways) were the two largest. Given the medicinal properties of PR, KEGG annotations related to the biosynthesis of active components were classified into ten major metabolic pathway categories, including carbohydrate, terpenoid and other secondary metabolite biosynthesis. Notably, carbohydrate metabolism contained the highest number of annotated genes (401 genes) (Figure S2D).

### 2.4. SSR Markers Development and Genetic Diversity Analysis

Three primer pairs were designed for each of the 43,217 SSR loci. Primer design was successful for 31,703 loci, whereas 11,514 loci (26.64%) were unsuitable for EST-SSR marker development due to short flanking sequences, despite containing SSRs (Table S6). From these, 100 SSR primer pairs were chosen for marker development (Table S7). Validation showed that 49 of 100 SSR primer pairs generated specific polymorphic bands (Table S8). Among the eight SSR locus types, p2 had the highest polymorphism rate at 65%, while the complex repeat type c* had the lowest at 20%.

The 49 polymorphic primers were further tested on 18 additional *Polygonatum* accessions (Table S9). Results showed that the 21 accessions yielded 446 polymorphic alleles. Number of alleles (Na) ranged from 4 to 17, with an average of 9.10. Effective number of alleles (Ne) averaged 4.70. Shannon’s information index (I) averaged 1.74. The expected heterozygosity (He) averaged 0.76. These metrics, with a Nei’s genetic identity (Nei’s I) index of 0.7748, collectively suggest substantial genetic diversity within the 21 *Polygonatum* accessions. The mean polymorphism information content (PIC) was 0.718, with 47 of the primer pairs qualifying as highly polymorphic markers (PIC > 0.5) [24,25]. Furthermore, the genetic differentiation coefficient (Fst) values ranged from 0.0449 to 0.8054. The average values of the inbreeding coefficient within subpopulations (Fis) and the overall inbreeding coefficient (Fit) were both positive.

### 2.5. Cluster Analysis

**Figure 3:**
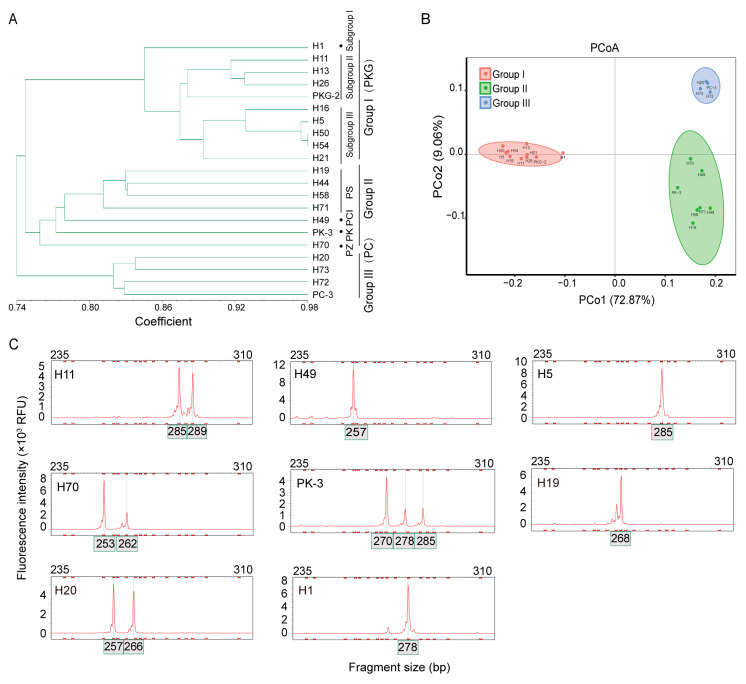
Genetic analysis of 21 *Polygonatum* accessions. (**A**) Phylogenetic tree of 21 *Polygonatum* accessions using 49 SSR markers; PS, *Polygonatum sibiricum*; PK, *P. kingianum*; PC, *P. cyrtonema*; PKG, *P. kingianum* var. *grandifolium*; PCI, *P. cirrhifolium*; PZ, *P. zanlanscianense*. (**B**) Principal coordinate analysis of 21 accessions; (**C**) Fluorescent electrophoresis images of FB-9 SSR markers in *Polygonatum* accessions.

Using the unweighted pair group method with arithmetic mean (UPGMA) method, a dendrogram was constructed for 21 accessions based on Nei’s I. The coefficient values had an average of 0.7817. At the individual level, the highest value (0.9799) was observed between accessions H50 and H54, indicating the closest genetic relationship. Conversely, the lowest value (0.7159) was detected between H1 and H70/H72, reflecting the most distant genetic relationship and substantial genetic divergence. When Nei’s I was set at 0.75, the accessions were classified into three clusters (Groups I, II, and III) (Figure 3A).

Group I consisted of 10 accessions, all originating from Sichuan Province in southwestern China. The average Nei’s I within this group was 0.88, with a minimum of 0.76, indicating a relatively homogeneous cluster. Accessions in this group exhibited irregular leaf arrangement patterns and were identified as PKG. At a genetic identity coefficient threshold of 0.87, the resources in Group I could be further subdivided into three subgroups. Subgroup II and III comprised accessions originating from northeastern and southern Sichuan, respectively. Furthermore, the subdivision of Group I was supported by seven molecular markers: FB-4, FB-8, FB-9, FB-57, FB-42, FB-60, and FB-98 (Table 1), the first six of which are associated with carbohydrate metabolism.

Group II consisted of seven accessions (H19, H44, H58, H71, H49, PK-3, and H70), with a wider geographic distribution in southwestern and central-southern China. The average Nei’s I was 0.78, with a minimum of 0.75. All accessions in Group II displayed whorled leaf arrangements and included species such as PK, PCI, PS, and PZ (*Polygonatum zanlanscianense* Pamp.). Group III contained four accessions (H20, H73, H72, and PC-3), which exhibited alternate leaf arrangements and were identified as PC. Principal coordinate analysis (PCoA) was also performed on the 21 *Polygonatum* resources, and the results were highly consistent with the cluster analysis (Figure 3B).

### 2.6. Comprehensive Evaluation of Group II Based on Phenotypic Traits and SSR Markers

**Figure 4:**
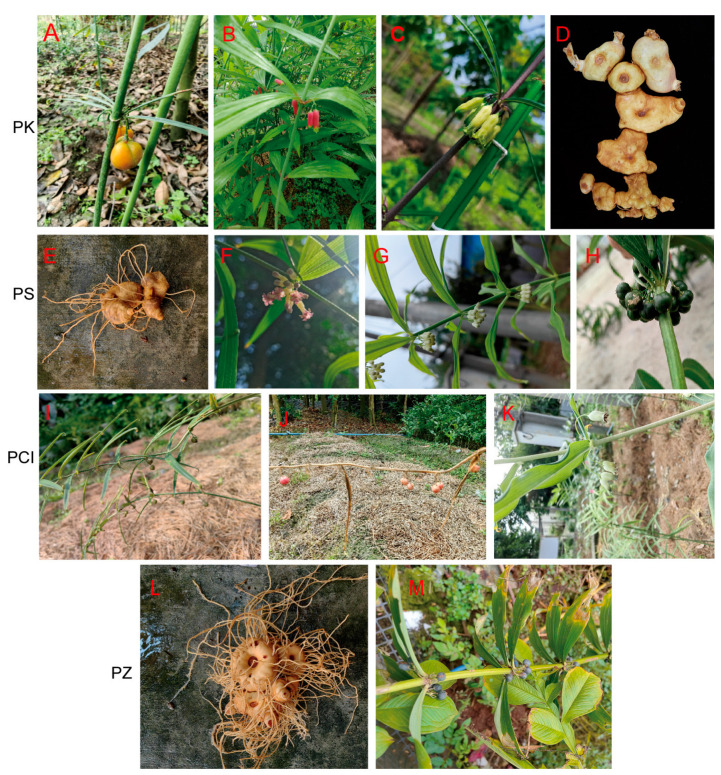
Morphological images of four *Polygonatum* germplasm resources (Group II). (**A**) Fruit of *P. kingianum*; (**B**) Red flower of *P. kingianum*; (**C**) Yellow-green flower of *P. kingianum*; (**D**) rhizome of *P. kingianum*; (**E**) rhizome of *P. sibiricum*; (**F**) Flower of *P. sibiricum* (H58); (**G**) Flower of *P. sibiricum* (H14); (**H**) Fruit of *P. sibiricum*; (**I**) Immature fruit of *P. cirrhifolium*; (**J**) Mature fruit of *P. cirrhifolium*; (**K**) Flower of *P. cirrhifolium*; (**L**) rhizome of *P. zanlanscianense*; (**M**) Leaf and mature fruit of *P. zanlanscianense*.

Significant morphological variation was observed among the resources of Group II, except for the highly similar pair H19 and H44. Based on a genetic identity coefficient of 0.80, this group could be further divided into four subgroups. H58 exhibited distinct differences from H19 and H44 (both PS) in floral morphology, size, and coloration. Compared with H9 (PK), the fruits of H70 are smaller and dark green, with round rhizomes that have a bitter taste, making it unsuitable as medicine. H49 plants are shorter, with significant differences in floral morphology, size, and coloration. The fruits of H71 are smaller and dark green (Figure 4). Based on morphology and molecular markers, H58 and H71 are identified as PS, H49 as PCI, and H70 as PZ. Interestingly, we found that three novel molecular markers (FB-5, FB-9, and FB-49) were capable of distinguishing the six *Polygonatum* species (Table 1). All three corresponding unigenes are associated with carbohydrate metabolism. Above, the FB-9 marker effectively differentiates among all six *Polygonatum* species and reveals intraspecific variation within the PKG (Figure 3C). Clustering analysis was performed again on 21 *Polygonatum* accessions utilizing nine selected molecular markers (FB-5, FB-9, FB-49, FB-4, FB-8, FB-57, FB-42, FB-60, and FB-98) (Figure S3). The results confirmed that these markers effectively differentiate among the six *Polygonatum* species and categorizing the intraspecific variation within PKG.

## 3. Discussion

### 3.1. Development of SSR Markers in Medicinal Polygonatum Species

In recent years, the declining cost of transcriptome sequencing has facilitated comprehensive acquisition of EST-SSRs, which can more efficiently enhance the management of germplasm collections relative to genome-derived SSRs by minimizing issues such as redundancy, mislabeling, and spelling errors [26]. Previous studies have successfully developed SSR markers for species within the genus *Polygonatum*. For instance, Pan et al. generated EST-SSR markers from the transcriptome of *P. odoratum* and demonstrated that specific primers could differentiate multiple *Polygonatum* species [11]. Similarly, SSR markers have been developed for *P. cyrtonema* [27], and their cross-species transferability within the genus has been explored [28]. These studies underscore the efficacy of SSR markers for lineage identification in *Polygonatum*. Nevertheless, these previous efforts primarily concentrated on the development or use of SSR markers for the identification of individual *Polygonatum* species and their intraspecific variations. A thorough examination of genetic diversity and species differentiation within MPs, utilizing an extensive array of newly developed EST-SSR markers, has yet to be adequately conducted. In the present study, transcriptome sequencing of three MPs facilitated the identification of 47,287 SSR motifs and the development of 31,703 SSR markers. These markers exhibit substantial potential for future applications in the identification and selective breeding of high-quality medicinal *Polygonatum* germplasms.

Furthermore, various research methodologies demonstrate considerable differences in the efficiency of SSR development. Based on the genome of PO, 153,753 SSR motifs were identified, far exceeding those identified in this study [28]. However, the number of polymorphic markers obtained from random primer screening was comparable: the genomic study obtained 54 polymorphic SSR markers from 100 primer pairs, whereas the transcriptome-based approach in this study obtained 49 polymorphic markers from 100 primer pairs. In contrast, based on transcriptome data from a single species (PO), only 30.88% of markers exhibited polymorphism upon validation [11]. Similarly, only 10% of SSR primer pairs showed polymorphism in PC [27]. Therefore, the more comprehensive transcriptome assembly constructed in this study, which incorporates multiple species, likely facilitated the detection of a greater number of low-abundance transcripts containing SSRs, thereby resulting in the identification of a higher number of polymorphic markers.

Concerning SSR frequency, the rate in MPs (23.79%) was lower than that in PO (29.47%) but higher than in *Medicago sativa* (21.64%) [18] and *Anoectochilus emeiensis* (Orchidaceae) (9.88%) [29]. This variation may be attributed to species-specific characteristics, reflecting the relatively abundant repetitive sequences in *Polygonatum* plants.

### 3.2. Number and Types of SSR Markers

The majority of EST-SSRs in plants are composed of SNRs and DNRs. In medicinal plants, dinucleotide repeats are chiefly observed in *Ilex asprella* [17], *Euphorbia fischeriana*, and *Euphorbia ebracteolata* [30], whereas trinucleotide repeats are more prevalent in *Anacardium occidentale* L. [24] and *Anoectochilus emeiensis* (Orchidaceae) [29]. Contrastingly, the present study identified an unusually high frequency of SNRs in MPs, accounting for 59.45%, which surpasses the proportions reported in PO (44.44%) [11] and yam (*Dioscorea* spp.) (50.73%) [31]. These SNRs were predominantly composed of A/T motifs, potentially indicating a nucleotide composition bias within the SSRs of MPs [23]. This bias may be attributed to two factors: the abundance of polyA/T structures, and the inherent tendency of A/T-rich repeat sequences to undergo DNA melting more easily. Beyond SNRs, DNRs constituted the largest fraction of the remaining polynucleotide SSRs (25.52%), a pattern consistent with sequencing data from PO [11]. Among DNRs, the AG/CT motif was the most abundant (16.73%), aligning with observations reported in PS [32]. This prevalence may be explained by its codon assignments, as AG/CT motifs frequently translate to the amino acids Ala and Leu, which occur more frequently in proteins than other amino acids. Regarding trinucleotide repeats, motifs such as AAG/CTT, AAT/ATT, and AGG/CCT each accounted for more than 2% of total SSRs, with AAT/ATT (2.72%) being the most prevalent. This distribution mirrors the trinucleotide repeat profiles reported in *Indigofera pseudotinctoria* [33] and various *Ipomoea* species [34]. Collectively, these findings reaffirm that simple repeat types constitute the vast majority of SSRs identified in plants. Furthermore, a substantial proportion (8.19%) of the SSR loci were identified as compound types c and c*. Among the nine core SSR markers selected in this study, four were compound SSR locus types (Table 1). Although most current efforts in molecular marker development have focused on simple SSRs [17,18,19], the findings of this study underscore the potential significance of developing complex SSR markers, which may not only play an important role in the biosynthesis of secondary metabolites but also hold great potential for variety identification in MPs.

### 3.3. Functional Annotation of the SSR-Containing Unigenes

Development of molecular markers tightly linked to genes involved in bioactive compound synthesis supports functional genomics and genetic breeding of *Polygonatum*. In this study, sequences harboring SSR loci were annotated by aligning 36,842 SSR-containing sequences against seven databases (NR, KOG, GO, SwissProt, NT, Pfam, and KO). The NR database yielded the most annotations (12,383 sequences), with the highest matches to *Asparagus officinalis*, likely due to their shared family (Liliaceae). GO classification assigned the EST-SSR sequences to three main categories and 41 subcategories, showing a distribution similar to that reported for safflower SSRs [35]. KEGG analysis revealed 222 annotated pathways, with carbohydrate metabolism contained the most genes (401 entries), consistent with polysaccharides being the major bioactive constituents in *Polygonatum*.

### 3.4. Genetic Diversity Parameters of SSR Loci

Research on genetic diversity is of great importance for elucidating species origin and evolutionary processes, as well as for the development of core germplasm resources [36,37]. In the study, genetic diversity parameters were evaluated for 21 *Polygonatum* resources across 49 SSR loci, yielding an average observed Na of 9.10, Ne of 4.70, Ho of 0.393, He of 0.763, mean PIC of 0.718, and average I of 1.74. Collectively, these metrics indicate substantial genetic differentiation and a high degree of genetic variability within the *Polygonatum* resources. Furthermore, the FST was employed to evaluate genetic divergence among populations [38]. The Nm quantifies the influence of gene flow mediated by migrating individuals on population genetic variability; Fis denotes the inbreeding coefficient of individuals relative to their subpopulations; and Fit represents the inbreeding coefficient of individuals relative to the total population [17]. In this investigation, the mean Fst was 0.307 (exceeding the threshold of 0.25), and the mean Nm was 0.876. Combined with positive mean values of Fis and Fit, these findings suggest concurrent effects of inbreeding within populations and significant genetic differentiation among populations, indicating the possible existence of geographic or reproductive isolation mechanisms.

### 3.5. Cluster Analysis and Its Correlation with Phenotypic

In studies examining plant morphological diversity, phenotypic polymorphism is often relatively limited, which consequently impacts the precision of plant clustering based on this approach [27]. The leaf arrangement (phyllotaxy) in *Polygonatum* species exhibits evolutionary instability [38]. Moreover, the similarity in the morphological characteristics of *Polygonatum* species, especially in the rhizome, frequently leads to misidentification in medicinal applications [15]. For instance, distinguishing between the aerial parts of PC and PO [11], or between the underground parts of PC and PKG [15], is difficult. Therefore, classification of *Polygonatum* species solely on morphological characteristics has proven to be challenging [28]. EST-SSR markers from PO can differentiate six *Polygonatum* species [11], while 17 polymorphic markers separated six wild *Luculia Yunnan Provinceensis* into two geographically distinct groups [39]. Molecular markers proved effective in detecting subtle genetic variation that is often undetectable through morphological assessment [40,41]. Thus, SSR markers offer greater suitability for assessing genetic diversity at both individual and population scales, in addition to providing enhanced efficiency and ease of application [16,17,18,19]. In summary, the integration of highly polymorphic molecular marker techniques with morphological analysis is necessary to elucidate the diversity of plant germplasms and to identify key genetic resources, thereby providing a scientific foundation for their conservation and breeding applications [42].

In the present study, genetic similarity coefficients were employed to classify 21 *Polygonatum* accessions into three groups (I, II, and III). This grouping exhibited a strong correlation with phyllotaxy, corresponding to irregular (alternate at both ends and whorled in the middle), whorled, and alternate leaf arrangements, respectively. PCoA further corroborated the observed genetic variation among the *Polygonatum* germplasms. Within Group I (all classified as PKG), subgroup I consisted solely of that a single wild accession H1, suggesting either a divergent ancestral origin or the retention of more primitive genetic traits. Subgroups II and III, originating from southern and northeastern Sichuan respectively, exhibited discernible regionality. Group II presented greater complexity. Through combined morphological and molecular marker analyses, accessions H58 and H71 were identified as PS, H49 as PCI, and H70 as PZ. Notably, PCI is recognized as a medicinal species, whereas the rhizome of PZ is bitter and deemed unsuitable for medicine [3].

### 3.6. Screening of SSR Markers

Based on amplification information of SSR loci, each of three markers (FB-5, FB-9, and FB-49) among 49 polymorphic markers can specifically discriminate six different *Polygonatum* resources: PS, PK, PC, PKG, PCI, and PZ. Considerable morphological variation exists within PKG, such as plant height, stem diameter, stem count, and phyllotaxy [4]. Furthermore, each of the seven SSR markers FB-4, FB-8, FB-9, FB-57, FB-42, FB-60, and FB-98 can distinguish the three subgroups of PKG, further demonstrating the effectiveness of SSR markers for intraspecific identification. Importantly, cluster analysis using the above nine selected SSR markers across 21 *Polygonatum* accessions yielded a result comparable to that obtained with the full set of 49 markers. The underprediction error (allelic dropout or missing data) was 3.21%, and the overprediction error (allele size discrepancy >3 bp between replicates) was 12.24%. These errors likely stem from PCR amplification artifacts and variations in fluorescent signal detection. A previous study reported that SSR markers derived from the *P. odoratum* genome failed to distinguish accessions by geographic origin or cultivar [28]. Notably, our findings reflect only the discriminatory power within the current germplasm; future studies with expanded sample sizes are necessary to validate the stability and generalizability of these markers. Together, these findings underscore the importance of developing a minimal set of precise and reliable molecular markers for effectively assessing genetic resources.

Polysaccharides are considered the principal bioactive constituents responsible for the therapeutic effects of medicinal *Polygonatum* species (MPs) [4,5,6,7,8,9,10]. Nevertheless, SSR markers associated with sugar metabolism have been minimally characterized within the genus *Polygonatum*. To date, only Pan et al. have reported a UDP-glucuronic acid transferase gene linked to the SSR marker YZ14; this gene encodes a glycosyltransferase implicated in the biosynthesis of polysaccharides [11]. In the present study, functional annotations of the nine core SSR markers revealed their association with carbohydrate metabolism, with the exception of FB-98. For instance, the genes corresponding to markers FB-5 and FB-49 were annotated as phosphoglucomutase/phosphomannomutase and α-galactosidase, respectively. Notably, the FB-9 marker not only distinguishs all six *Polygonatum* species but also differentiates intraspecific variation within PKG. The gene associated with this marker encodes UDP-glucose 4-epimerase, an isomerase essential for sugar metabolism and cell wall biosynthesis. The observed differential expression of this gene among three MPs further highlights its potential as a resource for future research into polysaccharide biosynthesis and genetic improvement of MPs. Overall, the integration of SSR markers with functional gene annotations provides a foundation for understanding the molecular mechanisms underlying polysaccharide biosynthesis in MPs and provides practical tools for marker-assisted selection in quality breeding.

## 4. Materials and Methods

### 4.1. Plant Materials

A total of 21 *Polygonatum* accessions were collected from four provinces in China, including 16 accessions from Sichuan, 3 accessions from Hunan, 1 accession from Yunnan, and 1 accessions from Chongqing. All accessions were uniformly cultivated at the Pidu Base of Sichuan Academy of Agricultural Characteristic Plants (Table 2). Fresh rhizome tissues were taken from each accession, washed with water, blotted dry, and stored at −80 °C for DNA extraction. Rhizomes of PC, PK, and PKG were collected for subsequent RNA extraction and transcriptome sequencing. Three biological replicates of the rhizome tissue were prepared.

### 4.2. SSR Identification and Functional Annotation of Unigenes

The MIcroSAtellite (MISA) program (http://pgrc.ipk-gatersleben.de/misa/ (accessed on 2 April 2025)) was employed to identify microsatellite motifs, with the minimum repeat thresholds for each unit size set as follows: 10 repeats for SNRs, 6 repeats for DNRs, 5 repeats for TNRs, TtNRs, PNRs, and HNRs. These threshold values were selected based on the default recommendations provided by the MISA software (version 1.0, IPK Gatersleben, Seeland, Germany) to ensure the development of reliable microsatellite markers [43]. Additionally, compound SSRs were defined as cases where the interval between two SSR sequences was less than 100 bp. To explore the potential functions of unigenes harboring SSR loci, the sequence reads were assembled into transcripts. Functional annotation of these unigenes was performed using databases such as NR, NT [44], Pfam [45], KOG [46], Swiss-Prot [47], KEGG [48], and GO [49], with the assistance of TBtools software (version 2.376, South China Agricultural University, Guangzhou, China).

### 4.3. Primer Design and Verification of EST-SSR Markers

Primers were designed with Primer3 software (version 2.3.5, San Diego, CA, USA) based on complementary sequences flanking SSR loci. Parameters were set as follows: primer length approximately 20 bp, Tm value 57–63 °C, and amplicon length 100–280 bp. For each predicted SSR locus, three pairs of primers were designed. A total of 100 SSR primer pairs were randomly selected from all primers we had designed based on different locus types. Specifically, 20 pairs were randomly chosen each for p2 and p3, respectively, and 10 pairs for each of the other types (Table S7). A universal M13 adapter sequence (TGTAAAACGACGGCCAGT) was added to the 5′ end of each forward primer before synthesis. Fluorescent dyes (FAM, HEX, TAMRA, ROX) were used for primer labeling, and fluorescence-based electrophoresis of the PCR products was performed on an ABI3730xl DNA sequencer (Applied Biosystems, Foster City, CA, USA).

Genomic DNA from 21 accessions was extracted using the CTAB method, and DNA quality was assessed using a UV spectrophotometer (Beijing New Technology Application Institute, Beijing, China). PCR amplification (Applied Biosystems, Foster City, CA, USA) was performed in a reaction mixture containing: 5 μL of 2X Taq plus PCR Master Mix, 0.1 μL of forward primer (10 μM), 0.3 μL of reverse primer (10 μM), 0.2 μL of M13 primer (10 μM), 1 μL of DNA template (30 ng/μL), and ddH_2_O was added to a final volume of 10 μL. The PCR program consisted of: pre-denaturation at 95 °C for 5 min; 10 cycles of denaturation at 94 °C for 30 s, annealing with temperature decreasing from 65 °C to 55 °C by 1 °C per cycle for 30 s, and extension at 72 °C for 40 s; followed by 25 cycles of denaturation at 94 °C for 30 s, annealing at 55 °C for 50 s, and extension at 72 °C for 40 s; a final extension at 72 °C for 7 min; and storage at 4 °C.

For capillary electrophoresis (Applied Biosystems, Foster City, CA, USA), a mixture containing 0.3 μL of PCR product, 0.5 μL of size standard, and 9.5 μL of deionized formamide (all from Applied Biosystems, Foster City, CA, USA) was loaded into a PCR plate (Axygen Scientific, Union City, CA, USA). The mixture was denatured at 95 °C for 5 min, chilled at 4 °C, centrifuged, and then loaded with 1× running buffer for detection.

### 4.4. Data Analysis

The raw data files from the sequencer were imported into GeneMarker 2.2.0 (SoftGenetics, State College, PA, USA) for analysis. Specific bands for each individual were recorded as band sizes (bp) to generate a locus information table and establish a raw data matrix. Using Popgene (version 1.32; F. C. Yeh, University of Alberta, Edmonton, AB, Canada) and GenAlEx software (version 6.501; Australian National University, Canberra, Australia), various genetic diversity indices for SSR loci and populations were calculated. These included the number of observed alleles (Na), effective alleles (Ne), Shannon’s information index (I), polymorphism information content (PIC), observed heterozygosity (Ho), expected heterozygosity (He), inbreeding coefficient within subpopulations (Fis), overall inbreeding coefficient (Fit), genetic differentiation coefficient (Fst), and gene flow (Nm, calculated as Nm = 0.25 × (1 − Fst)/Fst) [17].

A dendrogram of the 21 accessions was constructed using the UPGMA analysis in NTSYS-pc 2.10e (Applied Biostatistics Inc., New York, NY, USA). Nei’s genetic identity (Nei’s I) was also computed using NTSYS-pc 2.10e. Additionally, PCoA of the 21 *Polygonatum* resources was performed using the online tool available at https://magic.novogene.com/ (accessed on 15 December 2025).

## 5. Conclusions

In this study, a total of 43,217 SSR loci were identified from the rhizome transcriptomes of three MPs, leading to the development of 31,703 primers. Unigenes containing SSR loci were annotated against seven databases, with 46.83% successfully annotated. SNRs were the predominant SSR motif type, accounting for 59.45% of all identified motifs. From an initial screening of 100 molecular markers, 49 polymorphic SSR markers were selected, all of which exhibited high informativeness. Genetic diversity analysis revealed the presence of intraspecific inbreeding alongside pronounced interspecific differentiation among the 21 accessions, indicating a high overall level of genetic diversity. Cluster analysis categorized these 21 accessions into three principal groups. Furthermore, based on locus-specific information, three primer pairs were identified that can specifically discriminate among six *Polygonatum* species, and seven primer pairs were obtained for distinguishing intraspecific resources within PKG. Cluster analysis using these nine markers achieved a result comparable to that obtained with the full set of 49 markers. Collectively, these findings enhance the SSR marker repertoire available for *Polygonatum* species, offering valuable molecular tools for genetic characterization and functional gene discovery in medicinal *Polygonatum*, as well as providing technical support for the breeding of novel high-quality cultivars.

## Figures and Tables

**Table 1:** Characteristics of 9 primers for three MPs.

Locus Name	SSR	Forward Primer (5′-3′)	Reverse Primer (5′-3′)
Primers for determining species affiliation within a genus Polygonatum. *P. sibiricus*, *P. kingianum*, *P. cyrtonema*, *P. kingianum* var. grandifolium, *P. cirrhifolium* and *P. zanlanscianense*			
FB-5	(T)14aatttt(ATA)5	GGAGCTCACAAGACTGCACT	GCAAGTAACCAGCTTGAGCAG
FB-9	(GC)7(AC)14	CCCCCTGTAGGTTGATAGCC	CTTGCAGGTAAGTCCAGCCA
FB-49	(TC)6	ATTGCCAACGAATTGCTGCC	CCTCGTCCCATTCCTCCAAC
Primers for determining the three subgroups of *P. kingianum* var. *grandifolium*			
FB-4	(AAG)6acagcggaaaactgttgctgctttgtctgct(GGA)9	TCAGTCTCAGGCTGTTTTCCC	ATTGGCCTAGGAACTGCACC
FB-8	(CT)7cg(CT)6	GTGGAATTGTGCAGCTCAGC	TCGAGTGCTCCTCCCACTAA
FB-42	(GA)15	ACAGCGAGAGAGCATCAAGG	TCGCTTGCGAAGAAGAGAGG
FB-57	(TA)10	AGAAGAGCTGAACAAAAATGGCT	GGGGCTTCGCTGATGAGATT
FB-60	(TG)6	GGAGGGAAAATGGTGCATCC	GCTCATCAAGACCTAGCCCC
FB-98	(TCCGCC)6	ACTCCCGACTCCGAAAAACC	ACCGTGAACCCATCAACGAA

**Table 2:** Information on the 21 *Polygonatum* accessions.

Name	Species	Source	Longitude	Latitude	Types
H1	PKG	Xuyong City, Sichuan Province	105.44° E	28.16° N	Wild
H5	PKG	Luzhou City, Sichuan Province	105.44° E	28.87° N	Wild
PK-3	PK	Huili City, Sichuan Province	102.24° E	26.66° N	Cultivated
PKG-2	PKG	Dazhu City, Sichuan Province	107.20° E	30.74° N	Cultivated
H11	PKG	Dazhu City, Sichuan Province	107.00° E	30.51° N	Cultivated
H13	PKG	Yuechi City, Sichuan Province	106.44° E	30.54° N	Cultivated
H16	PKG	Dongxing District, Sichuan Province	105.08° E	29.60° N	Cultivated
H19	PS	Xiushan City, Chongqing Province	108.99° E	28.45° N	Cultivated
H20	PC	Meishan City, Sichuan Province	103.48° E	29.60° N	Cultivated
H21	PKG	Shuangliu District, Sichuan Province	103.92° E	30.58° N	Cultivated
H26	PKG	Shunqing District, Sichuan Province	106.09° E	30.80° N	Cultivated
PC-3	PC	Loudi City, Hunan Province	111.33° E	27.73° N	Cultivated
H44	PS	Loudi City, Hunan Province	111.13° E	27.64° N	Cultivated
H49	Unknown	Lijiang City, Yunnan Province	100.24° E	26.82° N	Cultivated
H50	PKG	Dongxing District, Sichuan Province	105.17° E	29.84° N	Cultivated
H54	PKG	Dongxing District, Sichuan Province	105.26° E	29.75° N	Cultivated
H58	Unknown	Xinhua City, Hunan Province	111.20° E	27.37° N	Wild
H70	Unknown	Chongzhou City, Sichuan Province	103.34° E	30.76° N	Wild
H71	Unknown	Shifang City, Sichuan Province	104.17° E	31.13° N	Wild
H72	PC	Shifang City, Sichuan Province	103.99° E	31.28° N	Cultivated
H73	PC	Shifang City, Sichuan Province	103.88° E	31.27° N	Cultivated

## Data Availability

The original contributions presented in this study are included in the article/Supplementary Materials. The raw sequence data reported in this paper have been deposited in the Genome Sequence Archive in National Genomics Data Center with accession number CRA039185 that are publicly accessible at https://ngdc.cncb.ac.cn/gsa (accessed on 26 February 2026). Further inquiries can be directed to the corresponding authors.

## Supplementary Materials

The following supporting information can be downloaded at: https://www.mdpi.com/article/10.3390/ijms27062632/s1.
