# Supplementary material for: Transcriptome-Wide Identification and Development of SSR Markers for Genetic Diversity Studies in Medicinal Polygonatum Species
Source: Int J Mol Sci. 2026 Mar 13;27(6):2632. doi: 10.3390/ijms27062632 (PMC13026351; doi:10.3390/ijms27062632)

International Journal of
*Molecular Sciences*

MDPI

*Article*

# Transcriptome-Wide Identification and Development of SSR Markers for Genetic Diversity Studies in Medicinal *Polygonatum* Species

**Wenjuan Huang [1], Hui Wang [1], Majin Yang [1], Changhua Ye [1], Zhen Li [1] and Shengfu Zhong [2,*]**

[1] Sichuan Academy of Agricultural Characteristic Plants, Chengdu 611730, China; hwjuan@scsaas.cn (W.H.); wanghui@scsaas.cn (H.W.); yangmj93@scsaas.cn (M.Y.); ye@scsaas.cn (C.Y.); lizhen@blooge.cn (Z.L.)
[2] Industrial Crops Research Institute, Sichuan Academy of Agricultural Sciences, Chengdu 610300, China
* Correspondence: zhongshengfu@scsaas.cn

## 1. Supplementary Figures and Tables

### 1.1. Supplementary Tables

**Supplementary Table S1.** Results of SSR search in transcriptome.

| Description | Statistics |
|---|---|
| Total number of sequences examined | 198,752 |
| Total size of examined sequences (bp) | 142,330,816 |
| Total number of identified SSRs | 47,287 |
| Number of SSR containing sequences | 36,842 |
| Proportion of unigenes with SSRs | 18.54 % |
| Number of sequences containing more than 1 SSR | 8057 |
| Percent GC (%) | 46.79 % |
| Number of SSR Locus types | 43,217 |
| Number of unigenes containing more than one locus | 5522 |
| Number of unigenes containing only one SSR locus | 31,320 |
| Number of unigenes containing two or more SSR motif types | 2918 |

**Supplementary Table S2.** SSR loci position analysis in unigenes.

**Supplementary Table S3.** Constitution of different length SSR motif types in transcriptome.

| Motif types | Number Of repeat types | Number of SSR motifs |
|---|---|---|
| SNRs | 2 | 28,114 |
| DNRs | 4 | 12,068 |
| TNRs | 10 | 6404 |
| TtNRs | 28 | 439 |
| PNRs | 28 | 121 |
| HNRs | 69 | 141 |
| Total | 141 | 47,287 |

Note: SNRs, mono-nucleotide repeat types; DNRs, di-nucleotide repeat types; TNRs, tri-nucleotide repeat types; TtNRs, tetra-nucleotide repeat types; PNRs, penta-nucleotide repeat types; HNRs, hexa-nucleotide repeat types.

**Supplementary Table S4.** The distribution and constitution of SNRs, DNRs, and TNRs.

| Repeats | 5 | 6 | 7 | 8 | 9 | 10 | 11 | 12 | 13 | 14 | 15 | 16-46 | Total | Rate within repeats/ % | Rate in total SSRs/% |
|---|---|---|---|---|---|---|---|---|---|---|---|---|---|---|---|
| A/T | - | - | - | - | - | 11,236 | 5,422 | 3,399 | 2,051 | 1,428 | 1,058 | 3,371 | 27,965 | 99.47% | 59.14% |
| C/G | - | - | - | - | - | 48 | 25 | 21 | 20 | 15 | 7 | 13 | 149 | 0.53% | 0.32% |
| AC/GT | - | 674 | 416 | 316 | 177 | 110 | 98 | 60 | 11 | 22 | 9 | 91 | 1,984 | 16.44% | 4.20% |
| AG/CT | - | 2,360 | 1,434 | 978 | 686 | 482 | 526 | 403 | 140 | 146 | 132 | 622 | 7,909 | 65.54% | 16.73% |
| AT/AT | - | 760 | 383 | 269 | 178 | 127 | 161 | 237 | | | | | 2,115 | 17.53% | 4.47% |
| CG/CG | - | 40 | 15 | 4 | 1 | | | | | | | | 60 | 0.50% | 0.13% |
| AAC/GTT | 211 | 91 | 54 | 35 | 4 | 3 | 3 | | 1 | | | | 402 | 6.28% | 0.85% |
| AAG/CTT | 580 | 251 | 135 | 66 | 12 | 11 | 9 | 3 | 2 | 3 | | | 1,073 | 16.76% | 2.27% |
| AAT/ATT | 578 | 299 | 236 | 173 | | | | | | | | | 1,286 | 20.08% | 2.72% |
| ACC/GGT | 188 | 68 | 52 | 32 | 4 | 4 | 5 | | 1 | 1 | | | 355 | 5.54% | 0.75% |
| ACG/CGT | 83 | 36 | 16 | 7 | 1 | | | 1 | | | | | 144 | 2.25% | 0.30% |
| ACT/AGT | 83 | 23 | 9 | 6 | 1 | 2 | | | 1 | | 1 | | 126 | 1.97% | 0.27% |
| AGC/CTG | 481 | 204 | 109 | 67 | 9 | 9 | 4 | 4 | 1 | | | | 888 | 13.87% | 1.88% |
| AGG/CCT | 503 | 227 | 126 | 84 | 17 | 6 | 3 | 4 | 1 | | | | 971 | 15.16% | 2.05% |
| ATC/ATG | 360 | 144 | 61 | 29 | 10 | 7 | 4 | 2 | | 2 | | | 619 | 9.67% | 1.31% |
| CCG/CGG | 304 | 132 | 59 | 45 | | | | | | | | | 540 | 8.43% | 1.14% |

**Supplementary Table S5.** Constitution of SSR locus types in transcriptome.

| SSR locus types | Number of SSR loci | Rate of SSR loci | Number of unigenes |
|---|---|---|---|
| p1 | 25,014 | 57.88% | 22540 |
| p2 | 8767 | 20.29% | 8359 |
| p3 | 5323 | 12.32% | 5060 |
| p4 | 354 | 0.82% | 351 |
| p5 | 97 | 0.22% | 96 |
| p6 | 123 | 0.28% | 122 |
| c | 3434 | 7.95% | 3355 |
| c* | 105 | 0.24% | 103 |

Note: p1, mono-nucleotide repeat type; p2, di-nucleotide repeat type; p3, tri-nucleotide repeat type; p4, tetra-nucleotide repeat type; p5, penta-nucleotide repeat type; p6, hexa-nucleotide repeat type; c, complex repeat type; c*, interrupted complex type.

**Supplementary Table S6.** Primer design and related information for unigenes containing SSR loci.

**Supplementary Table S7.** Marker information of 100 SSR loci screening in three MPs.

**Supplementary Table S8.** Amplification information of 49 SSR loci.

**Supplementary Table S9.** Genetic diversity parameters of 49 SSR loci in 21 *Polygonatum* accessions.

| Locus | Na | Ne | I | Ho | He | Fis | Fit | Fst | Nm | PIC |
|---|---|---|---|---|---|---|---|---|---|---|
| FB-2 | 10 | 6.9677 | 2.0889 | 0.2778 | 0.8810 | 0.5973 | 0.6525 | 0.1370 | 1.5749 | 0.8406 |
| FB-4 | 8 | 3.8855 | 1.6203 | 0.7619 | 0.7607 | -0.0590 | 0.0376 | 0.0912 | 2.4911 | 0.7080 |

| | | | | | | | | | | |
|---|---|---|---|---|---|---|---|---|---|---|
| FB-5 | 13 | 6.7477 | 2.1783 | 0.5263 | 0.8748 | 0.1640 | 0.4790 | 0.3768 | 0.4136 | 0.8357 |
| FB-8 | 11 | 8.4808 | 2.2389 | 0.4762 | 0.9036 | 0.4500 | 0.5556 | 0.1920 | 1.0518 | 0.8704 |
| FB-9 | 15 | 9.5870 | 2.4784 | 0.5714 | 0.9175 | 0.1510 | 0.2750 | 0.1461 | 1.4612 | 0.8874 |
| FB-10 | 10 | 8.1124 | 2.1758 | 0.5263 | 0.9004 | 0.4673 | 0.6192 | 0.2851 | 0.6270 | 0.8640 |
| FB-11 | 4 | 2.1421 | 0.9887 | 0.3571 | 0.5529 | 0.2697 | 0.3025 | 0.0449 | 5.3162 | 0.4874 |
| FB-13 | 6 | 3.2190 | 1.4334 | 0.2381 | 0.7062 | 0.2769 | 0.5529 | 0.3817 | 0.4049 | 0.6564 |
| FB-15 | 9 | 4.6915 | 1.8137 | 0.1905 | 0.8060 | 0.5236 | 0.7189 | 0.4098 | 0.3600 | 0.7610 |
| FB-17 | 5 | 2.6316 | 1.2052 | 0.1000 | 0.6359 | 0.5714 | 0.8364 | 0.6182 | 0.1544 | 0.5711 |
| FB-18 | 9 | 7.0560 | 2.0686 | 0.1429 | 0.8792 | 0.8392 | 0.8815 | 0.2630 | 0.7004 | 0.8428 |
| FB-19 | 9 | 3.0745 | 1.5577 | 0.1765 | 0.6952 | 0.6203 | 0.8014 | 0.4771 | 0.2740 | 0.6484 |
| FB-21 | 7 | 3.2523 | 1.5013 | 0.2632 | 0.7112 | 0.4725 | 0.6198 | 0.2792 | 0.6455 | 0.6618 |
| FB-24 | 4 | 2.9302 | 1.1745 | 0.1429 | 0.6748 | -0.1285 | 0.7804 | 0.8054 | 0.0604 | 0.5930 |
| FB-25 | 5 | 2.7055 | 1.2217 | 0.4286 | 0.6458 | -0.3770 | 0.2901 | 0.4845 | 0.2660 | 0.5858 |
| FB-26 | 10 | 2.9797 | 1.5714 | 0.1905 | 0.6806 | 0.5972 | 0.7427 | 0.3612 | 0.4422 | 0.6420 |
| FB-27 | 5 | 4.2000 | 1.5054 | 0.7619 | 0.7805 | -0.2885 | 0.0066 | 0.2290 | 0.8416 | 0.7219 |
| FB-29 | 13 | 4.6421 | 2.0530 | 0.4286 | 0.8037 | 0.1702 | 0.4108 | 0.2899 | 0.6125 | 0.7719 |
| FB-31 | 13 | 4.4545 | 1.9837 | 0.8571 | 0.7944 | -0.3053 | -0.0552 | 0.1916 | 1.0547 | 0.7591 |
| FB-35 | 9 | 6.0411 | 1.9796 | 0.7143 | 0.8548 | -0.0714 | 0.1684 | 0.2238 | 0.8671 | 0.8156 |
| FB-40 | 11 | 5.6538 | 2.0160 | 0.4286 | 0.8432 | 0.2317 | 0.4488 | 0.2826 | 0.6347 | 0.8051 |
| FB-42 | 8 | 5.2632 | 1.8229 | 0.4500 | 0.8308 | 0.3064 | 0.5401 | 0.3370 | 0.4918 | 0.7846 |
| FB-44 | 6 | 2.3333 | 1.1718 | 0.1905 | 0.5854 | 0.4196 | 0.6323 | 0.3664 | 0.4323 | 0.5356 |
| FB-46 | 12 | 7.1707 | 2.2235 | 0.5238 | 0.8815 | 0.2385 | 0.3885 | 0.1970 | 1.0190 | 0.8483 |

| | | | | | | | | | | |
|---|---|---|---|---|---|---|---|---|---|---|
| FB-47 | 12 | 7.5349 | 2.2161 | 0.2778 | 0.8921 | 0.5531 | 0.5980 | 0.1005 | 2.2375 | 0.8539 |
| FB-48 | 9 | 2.5974 | 1.4570 | 0.1500 | 0.6308 | 0.6601 | 0.7831 | 0.3619 | 0.4408 | 0.5965 |
| FB-49 | 11 | 7.7670 | 2.1894 | 0.3500 | 0.8936 | 0.6177 | 0.7103 | 0.2421 | 0.7825 | 0.8579 |
| FB-50 | 6 | 3.1056 | 1.3487 | 0.1429 | 0.6945 | 0.5703 | 0.7560 | 0.4321 | 0.3286 | 0.6309 |
| FB-52 | 11 | 4.0833 | 1.8591 | 0.9048 | 0.7735 | -0.2133 | -0.1065 | 0.0880 | 2.5895 | 0.7373 |
| FB-53 | 7 | 4.4100 | 1.6273 | 0.3810 | 0.7921 | 0.3471 | 0.4980 | 0.2311 | 0.8320 | 0.7388 |
| FB-55 | 17 | 5.1882 | 2.2484 | 0.5714 | 0.8269 | 0.1159 | 0.2625 | 0.1659 | 1.2573 | 0.7970 |
| FB-57 | 10 | 5.7273 | 1.9745 | 0.3333 | 0.8455 | 0.4881 | 0.5939 | 0.2067 | 0.9596 | 0.8059 |
| FB-58 | 10 | 4.5231 | 1.8210 | 0.4762 | 0.7979 | 0.2323 | 0.3336 | 0.1320 | 1.6446 | 0.7535 |
| FB-60 | 13 | 10.0000 | 2.4148 | 0.6500 | 0.9231 | 0.2727 | 0.3676 | 0.1305 | 1.6664 | 0.8916 |
| FB-69 | 13 | 4.6915 | 2.0779 | 0.1429 | 0.8060 | 0.7070 | 0.7928 | 0.2928 | 0.6038 | 0.7751 |
| FB-70 | 13 | 3.9200 | 1.9356 | 0.2857 | 0.7631 | 0.3952 | 0.5269 | 0.2177 | 0.8982 | 0.7312 |
| FB-73 | 10 | 6.3894 | 2.0344 | 0.5263 | 0.8663 | 0.1241 | 0.3321 | 0.2375 | 0.8028 | 0.8258 |
| FB-76 | 5 | 2.3710 | 1.0949 | 0.4762 | 0.5923 | -0.2760 | 0.4053 | 0.5339 | 0.2182 | 0.5284 |
| FB-77 | 8 | 4.7419 | 1.7736 | 0.5238 | 0.8084 | 0.1682 | 0.3668 | 0.2387 | 0.7972 | 0.7631 |
| FB-78 | 5 | 2.9205 | 1.2447 | 0.2857 | 0.6736 | -0.1430 | 0.4375 | 0.5079 | 0.2423 | 0.5952 |
| FB-84 | 10 | 5.3133 | 1.9716 | 0.2857 | 0.8316 | 0.5604 | 0.6790 | 0.2697 | 0.6769 | 0.7936 |
| FB-85 | 6 | 2.4915 | 1.1607 | 0.0952 | 0.6132 | 0.5345 | 0.7939 | 0.5573 | 0.1986 | 0.5366 |
| FB-86 | 8 | 2.2443 | 1.2309 | 0.2381 | 0.5679 | 0.1703 | 0.5897 | 0.5055 | 0.2446 | 0.5271 |
| FB-87 | 9 | 3.7383 | 1.7294 | 0.3500 | 0.7513 | 0.2847 | 0.4908 | 0.2882 | 0.6175 | 0.7123 |
| FB-90 | 10 | 3.1613 | 1.5351 | 0.3333 | 0.7003 | -0.0433 | 0.4015 | 0.4263 | 0.3364 | 0.6416 |
| FB-96 | 12 | 4.0833 | 1.8840 | 0.5714 | 0.7735 | -0.0388 | 0.2116 | 0.2410 | 0.7873 | 0.7374 |

| | | | | | | | | | | |
|---|---|---|---|---|---|---|---|---|---|---|
| FB-97 | 4 | 1.5527 | 0.7042 | 0.1053 | 0.3656 | 0.2016 | 0.6924 | 0.6147 | 0.1567 | 0.3299 |
| FB-98 | 9 | 5.2189 | 1.9084 | 0.6190 | 0.8281 | 0.0154 | 0.2450 | 0.2331 | 0.8223 | 0.7887 |
| FB-100 | 6 | 4.3024 | 1.5968 | 0.4762 | 0.7863 | 0.1369 | 0.4031 | 0.3084 | 0.5607 | 0.7325 |
| mean | 8.84 | 4.4234 | 1.6933 | 0.3787 | 0.7514 | 0.2563 | 0.4966 | 0.3202 | 0.8161 | 0.7054 |

Note: Na, number of alleles; Ne, effective number of alleles; I, Shannon's information index; PIC, polymorphism information content; Ho, observed heterozygosity; He, expected heterozygosity; Fis, inbreeding coefficient within subpopulations; Fit, overall inbreeding coefficient; Fst, genetic differentiation coefficient; Nm, gene flow (calculated as Nm = 0.25 × (1 - Fst) / Fst).

## *1.2. Supplementary Figures*

**Supplementary Figure S1.** Composition and distribution of amino acids encoded by p3-Type TNRs.

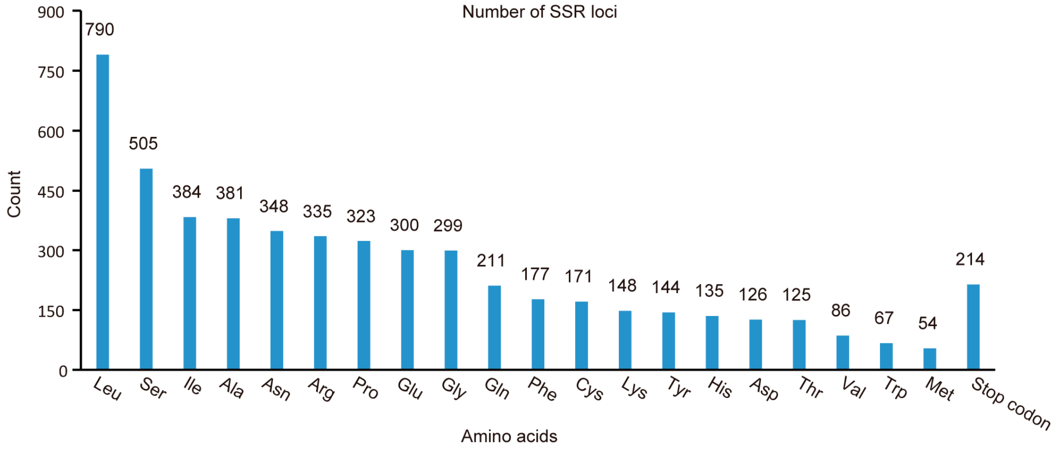


**Supplementary Figure S2.** Annotation of unigenes containing SSRs. (A) Distribution of GO classification of unigenes carrying SSRs; (B) Distribution of unigenes containing SSRs in KEGG functional categories; (C) Metabolic pathway mapping of unigenes containing SSRs based on KEGG BRITE classification; (D) Top species with the highest matched sequences in NR.

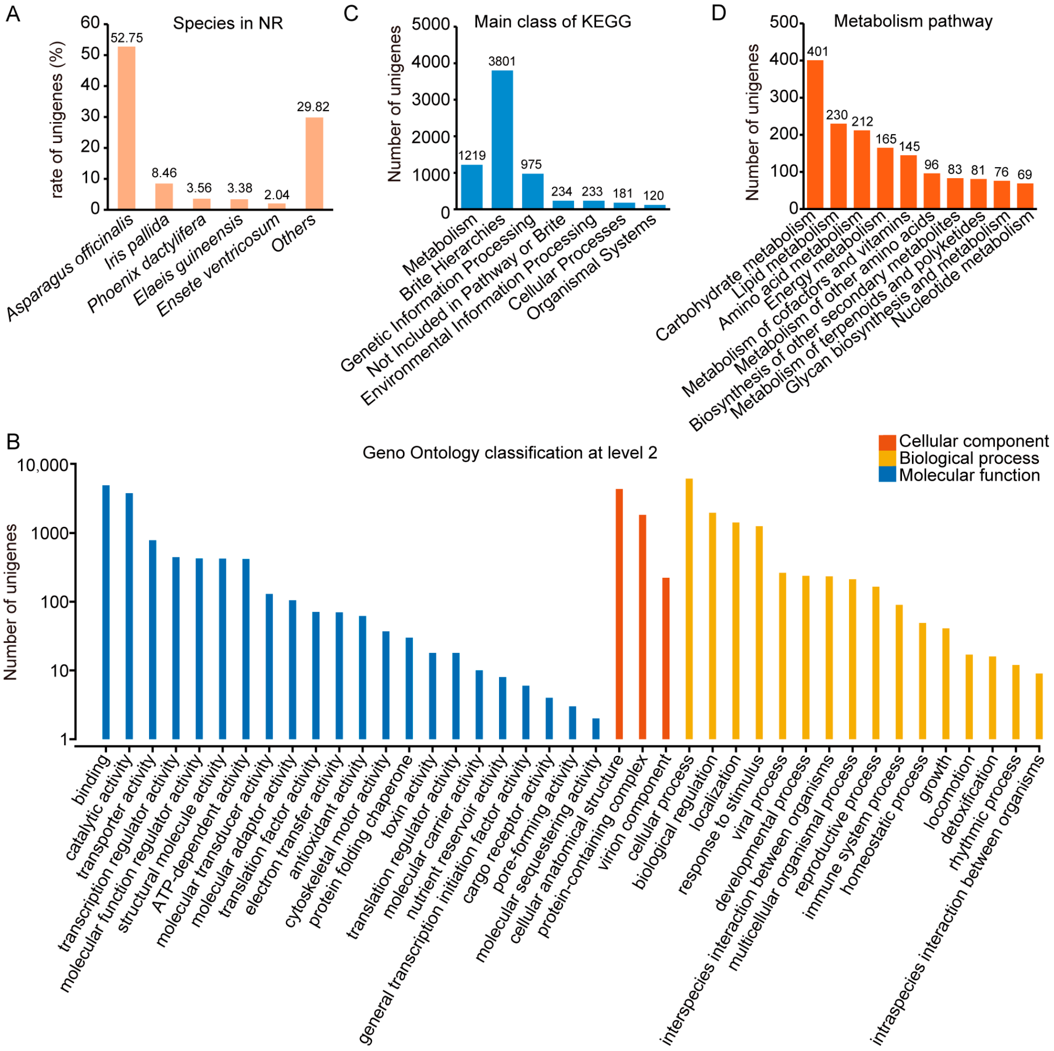

**Supplementary Figure S3.** Phylogenetic tree of 21 *Polygonatum* accessions using 9 SSR markers.

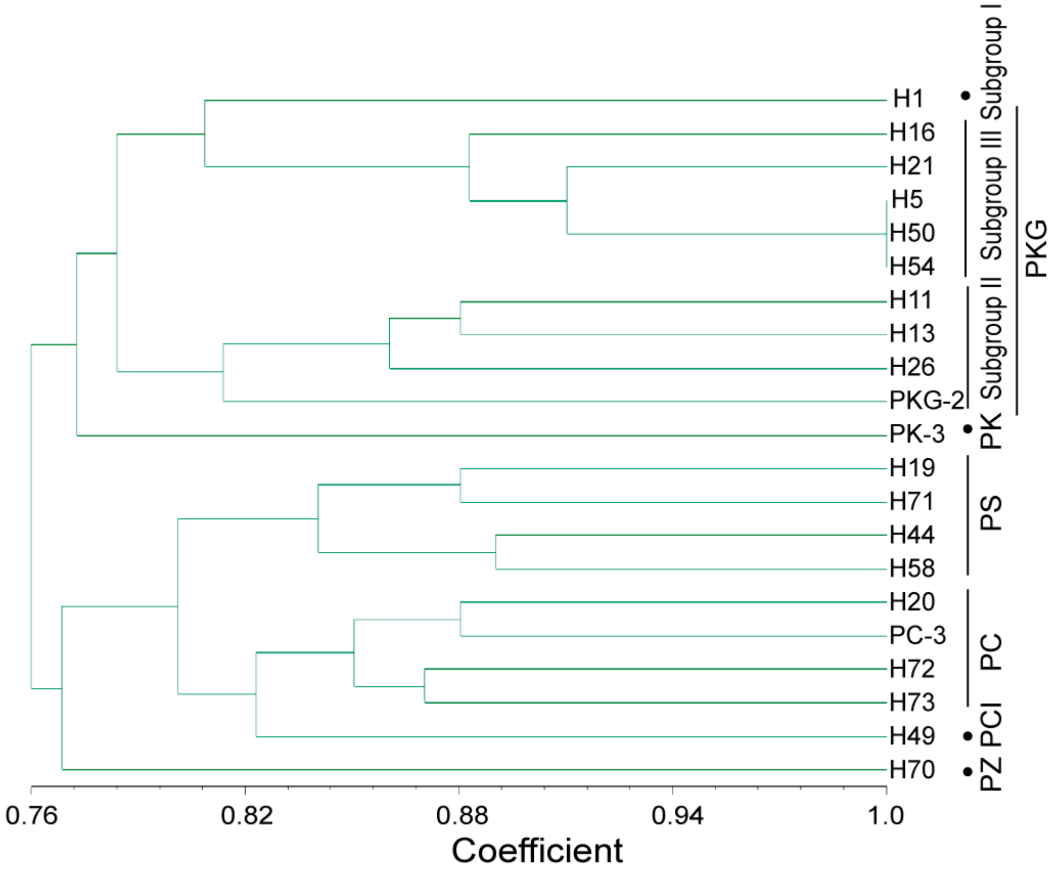

Supplement: Supplementary file 1 [file ijms-27-02632-s001.zip › Supplementary Materials.pdf]
